# Interaction of Caffeic Acid with SDS Micellar Aggregates

**DOI:** 10.3390/molecules24071204

**Published:** 2019-03-27

**Authors:** Antonio Cid, Oscar A. Moldes, Juan C. Mejuto, Jesus Simal-Gandara

**Affiliations:** 1 Physical Chemistry Department, Faculty of Sciences, University of Vigo, 32004 Ourense, Spain; moldes@uvigo.es (O.A.M.); xmejuto@uvigo.es (J.C.M.); 2 LAQV-UCIBIO, REQUIMTE, Departamento de Química, Faculdade de Ciências e Tecnología, Universidade NOVA de Lisboa, 2829-516 Caparica, Portugal; 3 Nutrition and Bromatology Group, Department of Analytical and Food Chemistry, Faculty of Food Science and Technology, University of Vigo, Ourense Campus, 32004 Ourense, Spain

**Keywords:** caffeic acid, SDS, micellization, critical micelle concentration, anionic amphiphiles, food additives

## Abstract

Micellar systems consisting of a surfactant and an additive such as an organic salt or an acid usually self-organize as a series of worm-like micelles that ultimately form a micellar network. The nature of the additive influences micellar structure and properties such as aggregate lifetime. For ionic surfactants such as sodium dodecyl sulfate (SDS), CMC decreases with increasing temperature to a minimum in the low-temperature region beyond which it exhibits the opposite trend. The presence of additives in a surfactant micellar system also modifies monomer interactions in aggregates, thereby altering CMC and conductance. Because the standard deviation of β was always lower than 10%, its slight decrease with increasing temperature was not significant. However, the absolute value of Gibbs free enthalpy, a thermodynamic potential that can be used to calculate the maximum of reversible work, increased with increasing temperature and caffeic acid concentration. Micellization in the presence of caffeic acid was an endothermic process, which was entropically controlled. The enthalpy and enthropy positive values resulted from melting of “icebergs” or “flickering clusters” around the surfactant, leading to increased packing of hydrocarbon chains within the micellar core in a non-random manner. This can be possibly explained by caffeic acid governing the 3D matrix structure of water around the micellar aggregates. The fact that both enthalpy and entropy were positive testifies to the importance of hydrophobic interactions as a major driving force for micellization. Micellar systems allow the service life of some products to be extended without the need to increase the amounts of post-harvest storage preservatives used. If a surfactant is not an allowed ingredient or food additive, carefully washing it off before the product is consumed can avoid any associated risks. In this work, we examined the influence of temperature and SDS concentration on the properties of SDS–caffeic acid micellar systems. Micellar properties can be modified with various additives to develop new uses for micelles. This allows smaller amounts of additives to be used without detracting from their benefits.

## 1. Introduction

**Scheme 1:**
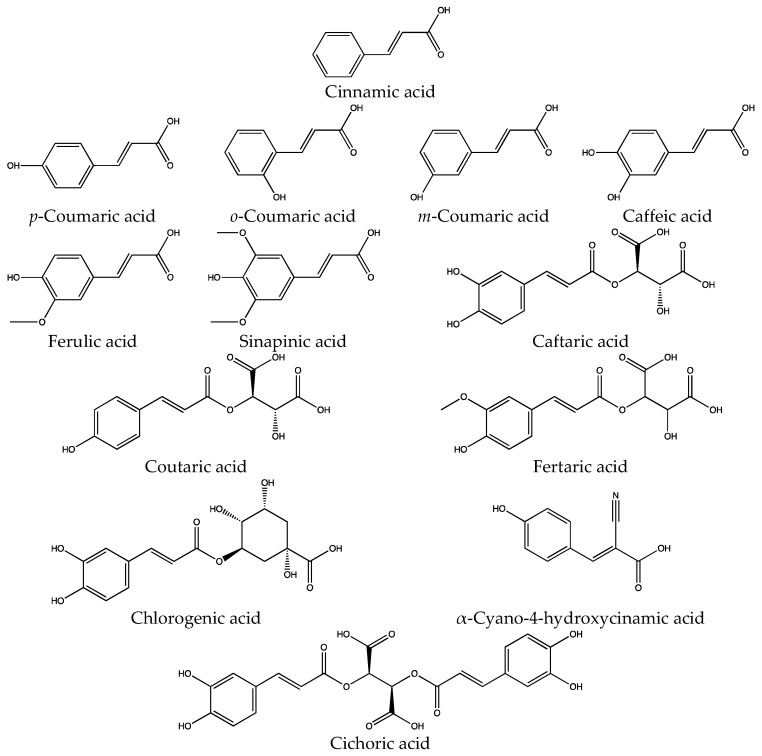
Cinnamic acid and hydroxycinnamic acid derivatives.

Hydroxycinnamic acids are phenolic compounds of the phenylpropanoid family that occur naturally as secondary metabolites in plants. A variety of these derivatives of cinnamic acid can in fact be found in foods, plant-derived products (e.g., beer, wine, olive oil, coffee), herbs, spices, cereals, legumes, fruits, nuts, and vegetables [1,2], even in mushrooms [3], in variable amounts depending on a number of factors including environmental conditions, cultivation technique and plant part [4]. Scheme 1 shows selected hydroxycinnamic acids. Because these phenolic compounds are ubiquitous in plants, they are usual components of the animal and human diet, albeit in widely variable proportions around the world. Also, although they can be absorbed in the gastrointestinal tract, they are partially excreted unchanged or in derivative forms via urine and faeces [5]. 

Phenols promote health and reduce disease risk, so they are especially relevant to animal and human health, pharmacology, food science and industrial production. For example, the health effects of Mediterranean diet have been widely ascribed to the consumption of phenolic compounds even though the biologically available amount of substances such as flavonoids in foods may be inadequate to account for their action and synergistic effects may be present [6]. Some major features of fruit production are strongly influenced by the presence of phenols. In fact, phenolic compounds influence fruit quality through a number of sensory properties such as flavour [7], colour [8] or texture [9]. Also, the outcome of storage and other manipulation processes [10,11] may be compromised by browning reactions involving phenols and resulting in undesirable colour or taste, or the loss of nutritional value, all of which can have adverse economic impacts.

Caffeic acid (CA) is an especially promising nutraceutical by virtue of its strong antioxidant effects [12,13,14]. Thus, CA modulates redox balance in cells and protects them from reactive oxygen species (ROS) forming in their metabolic reactions [15,16]. In fact, CA scavenges free radicals, thereby stopping oxidation; the effect is ascribed to its two hydroxyl groups and their position on the molecule facilitating hydrogen bonding interactions. As a result, CA has some potential health benefits especially prominent among which are anti-carcinogenic action [17,18,19], and cancer prevention and treatment. In addition, CA is an effective anti-mutagenic agent with activity against nitrosamines [20]. However, it remains as a potential carcinogen for humans on some hazard sheets [21]. In food technology, caffeic acid has proved effective to reduce aflatoxin biosynthesis during nut storage [22]. Obviously, high fat contents or the presence of salts can trigger degradation. Finally, because consumers are increasingly favouring naturally occurring products, compounds such as CA may find even wider use.

Micellar systems are colloids with useful properties for industrial and food applications. For example, they can be used to prepare isotropic, thermodynamically stable mixtures of polar and non-polar solvents to solubilize hydrophobic food-related substances such as aromas or all types of preservatives. Also, they can be used to trap unwanted chemicals such as degradation precursors or off-odour compounds. Sodium dodecyl sulphate (SDS), a surfactant that tends to aggregate into micelles, has been widely studied as a model for micellar structures. Whereas European legislation banned the use of SDS as a direct additive for food, the US-FDA has recognised it as a multi-purpose additive [23]. 

In this work, we expanded previous analyses of the influence of temperature and SDS concentration on various SDS–phenolic acid micellar systems [24,25]. Micellar properties can be modified by using various additives with a potential impact on micelle uses. For example, some additives may be used in smaller amounts without sacrificing their benefits. In this work, we examined the properties of micellar systems consisting of caffeic acid and SDS.

## 2. Results and Discussion

**Figure 1:**
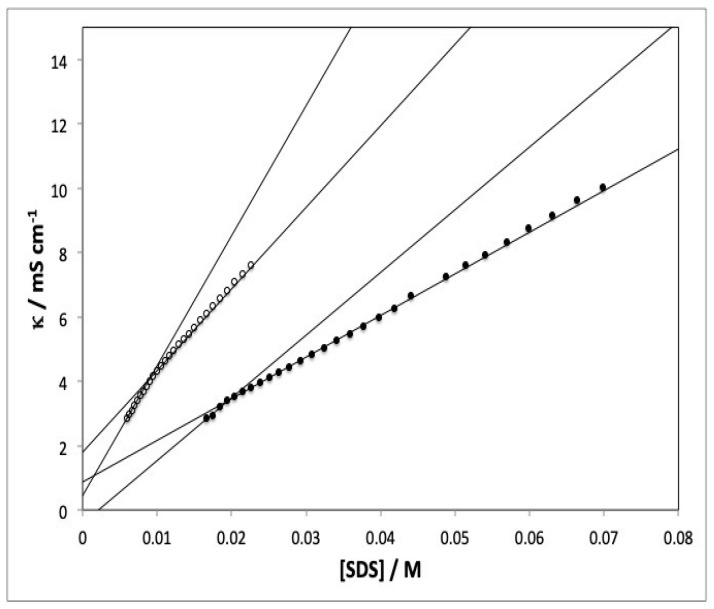
Typical specific conductance vs surfactant concentration plot for SDS in an aqueous colloid solution. [CA] = 3 × 10^−4^ M. Temperature: (●) 25 °C, (○) 55 °C.

The conductance of a solution is a linear function of the concentration of its components. Also, structural changes in a colloidal solution are known to modify their properties. For example, the formation of micellar aggregates interferes with the way electrons flow through the system. For this reason, conductance measurements allow the point where aggregates form to be accurately identified. The critical micelle concentration (CMC) of the SDS–caffeic acid micellar system was determined from specific conductance versus surfactant concentration curves. The graph exhibited two distinct regions differing in slope, namely: the surfactant monomer region and the micellar aggregate region (see Figure 1). The point where the two branches intersect is taken to represent the CMC of the surfactant. For SDS in water, CMC has been found to range from 8 to 8.3 m mol kg^−1^ [26].

Because interactions between monomers are affected by thermal agitation, which may facilitate or hinder aggregation depending on the nature of the particular molecules, CMC is strongly influenced by temperature [27,28]. For ionic surfactants such as SDS, CMC decreases with increasing temperature to a minimum in the low-temperature region beyond which it exhibits the opposite trend. The presence of additives in a surfactant micellar system also modifies monomer interactions in aggregates, thereby altering CMC and conductance. Table 1 illustrates the behaviour of the SDS–caffeic acid micellar system in this respect. 

**Figure 2:**
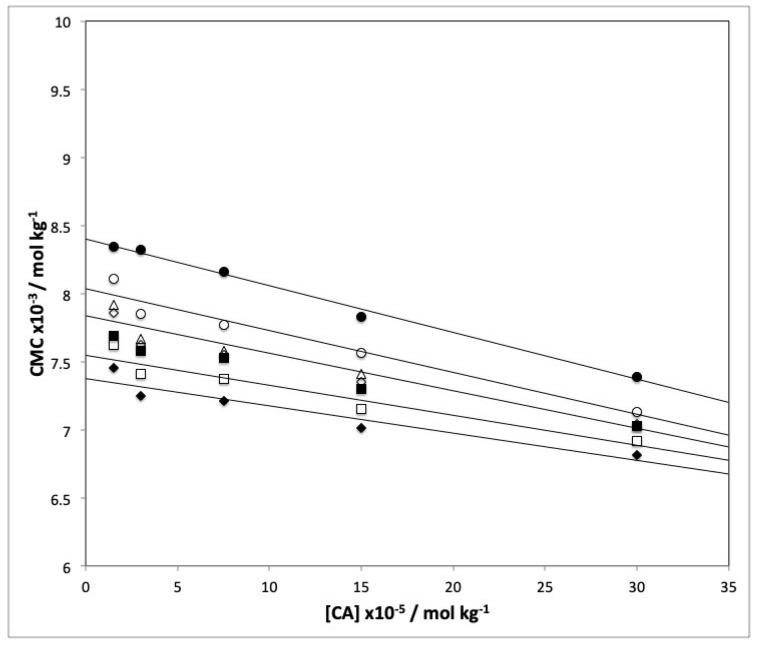
Influence of temperature and the caffeic acid concentration on CMC for the SDS–caffeic acid micellar system as determined from conductance measurements. Temperature: (●) 25 °C, (○) 30 °C, (△) 35 °C, (◇) 40 °C, (■) 45 °C, (☐) 50 °C and (◆) 55 °C.

Figure 2 illustrates the decrease in CMC with increasing caffeic acid concentration. CMC also decreased with increasing temperature at each CA level. *R*^2^ was invariably higher than 0.9232, so CMC was a linear function of the caffeic acid concentration. Indeed, the degree of micelle aggregation (β) was seemingly independent of the additive concentration and temperature. Because the standard deviation of β was always lower than 10%, its slight decrease with increasing temperature was not significant. We thus took β to be 0.68 ± 0.11.

As noted earlier, ∆*G*^0^*_m_* was calculated from the temperature-dependence of CMC (Equation (3)). As can be seen in Table 2, ∆*G*^0^*_m_* was negative throughout the temperature and CA concentration ranges; therefore, micellization was invariably spontaneous. It should be noted that the absolute value of ∆*G*^0^*_m_* increased with increasing temperature and CA concentration, probably as a result of a structural effect of caffeic acid on water.

**Figure 3:**
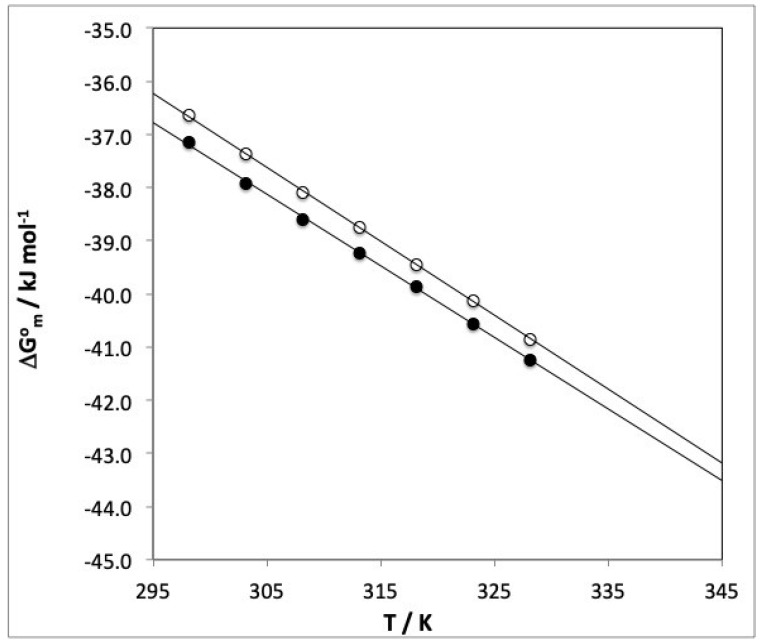
Influence of *T* (K) on ∆*G*^0^*_m_* (kJ mol^−1^) for SDS–CA. Caffeic acid concentration: (●) 30 × 10**^−^**^5^ mol kg**^−^**^1^, (○) 1.5 × 10**^−^**^5^ mol kg**^−^**^1^.

The influence of temperature on ∆*G*^0^*_m_* at different caffeic acid concentrations allowed us to to estimate the changes in standard micellization enthalpy (∆*H*^0^*_m_*) and standard micellization entropy (∆*S*^0^*_m_*). Figure 3 illustrates the influence of temperature on ∆*G*^0^*_m_* and Table 3 shows the values obtained at each CA concentration. *R*^2^ was always higher than 0.9985, so ∆*G*^0^*_m_* was linearly dependent on temperature.

Micellization in the presence of CA was an endothermic process and Δ*H*^0^_m_ decreased with increase in CA concentration, the two bearing a linear relationship (*R*^2^ = 0.9127). Also, Δ*S*^0^_m_ was positive, so the micellization process was entropically controlled. These positive values resulted from melting of “icebergs” or “flickering clusters” around the surfactant leading to increased packing of hydrocarbon chains within the micellar core in a non-random manner [28]. Δ*S*^0^_m_ also bore a linear relationship to the CA concentration (*R*^2^ = 0.8363). Raising such a concentration possibly led to caffeic acid governing the 3D matrix structure of water around the micellar aggregates. The fact that both Δ*H*^0^_m_ and Δ*S*^0^_m_ were positive testifies to the importance of hydrophobic interactions as a major driving force for micellization. 

## 3. Materials and Methods

### 3.1. Binding Mechanism

**Scheme 2:**
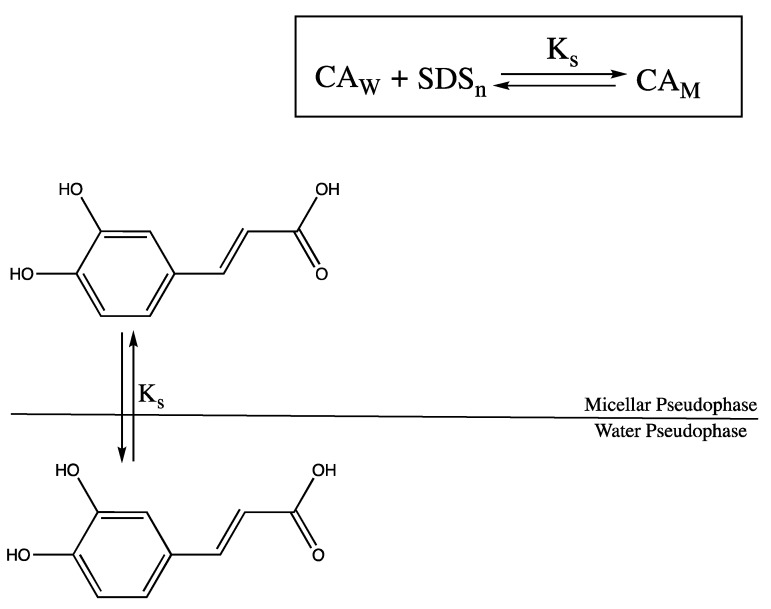
Proposed mechanism for CA binding to SDS micelles.

The proposed mechanism for CA binding to SDS micelles is depicted in Scheme 2. 

Based on the pseudo-phase model, interactions between SDS and CA are assumed to occur in different domains. In the scheme, *W* and *M* designate aqueous and micellar pseudo-phases, respectively. The binding constant is given by

(1) $$K_{S}=\frac{{\left[\mathrm{CA}\right]}_{M}}{[\mathrm{CA}{]}_{W}[{\mathrm{SDS}}_{n}]}$$

Also, the concentration of micellized surfactant, [SDS*_n_*], is assumed to be

(2) $$\left[{\mathrm{SDS}}_{n}\right]=\left[{\mathrm{SDS}}_{t}\right]-\mathrm{CMC}$$

where [SDS_t_] is the total concentration of SDS and CMC is the critical micelle concentration.

### 3.2. Conductivity Measurements

All reactants were supplied by Sigma Aldrich (Steinheim, Germany) and used without further purification. All solutions were prepared in distilled and deionized water (Adesco, Granollers, Spain). The experimental procedure is described in detail elsewere [24,25]. Conductance (κ) measurements accurate to within ±0.1% were made with a Crison GLP-32 conductimeter (Mettler-Toledo, L’Hospitalet de Llobregat, Spain) with a cell constant of 0.1 cm^−1^ that was calibrated with two different KCl standards (0.0100 KCl mol L^−1^, κ = 1413 μS cm^−1^ and 0.1000 KCL mol L^−1^, κ = 12.88 mS cm^−1^, both at 25 °C) supplied by Crison. The conductance of SDS solutions of variable concentration ranging from 0.1 to 0.004 mol L^−1^ was measured at a constant temperature and after addition of caffeic acid to a concentration of 0–7 ×·10^–5^ mol L^–1^ at 25–55 °C. A Techne TE-8D RB-5 thermostatic bath (Cole-Parmer, Staffordshire, UK) was used to keep samples at the desired temperature ±0.1 °C. Also, samples were magnetically stirred during measurements. 

### 3.3. CMC Determination

By virtue of their sensitivity and reproducibility, conductivity measurements provide an accurate method for determining CMC in anionic and cationic surfactants [29]. CMC is calculated from the point of intersection of two lines in conductivity versus surfactant concentration plot in two regions corresponding to surfactant monomers ([SDS] « CMC) and aggregates ([SDS] » CMC). Figure 1 illustrates the determination of CMC at two different temperatures and in the presence of CA as an additive.

### 3.4. Micellization Thermodynamics 

The molar standard Gibbs energy change for the micelle aggregation process can be calculated from the following equation [30]:

(3) $$∆G{{}^{\circ}}_{m}=\left(1+\beta \right)RT\ln X_{CMC}$$

where *T* is temperature, R the universal gas constant and *X*_CMC_ the critical micelle concentration as a mole fraction. β is the degree of micellization [31] and is calculated as the ratio between the slopes of conductance–concentration curves in the pre-micellar and post-micellar region (see Figure 1):

(4) $$\beta =\frac{S_{2}}{S_{1}}$$

The standard enthalpy and entropy of micellization (∆*H*^0^*_m_* and ∆*S*^0^*_m_*, respectively) can be used to calculate ∆*G*^0^*_m_* as follows:

(5) $$∆G{{}^{\circ}}_{m}=∆H{{}^{\circ}}_{m}-T∆S{{}^{\circ}}_{m}$$

## 4. Conclusions 

Micellar systems consisting of a surfactant and an additive such an organic salt or an acid usually self-organize into a series of worm-like micelles that ultimately form a micellar network. The nature of the additive influences some properties of the micellar structure including aggregate lifetime. For ionic surfactants such as SDS, CMC decreases with increasing temperature to a minimum in the low-temperature region beyond which it exhibits the opposite trend. The presence of additives in a surfactant micellar system also modifies monomer interactions in aggregates, thereby altering CMC and conductance. Because the standard deviation of β was always lower than 10%, its slight decrease with increasing temperature was not significant. However, the absolute value of Gibbs free enthalpy, a thermodynamic potential that can be used to calculate the maximum of reversible work, increased with increasing temperature and caffeic acid concentration. Micellization in the presence of caffeic acid was an endothermic process, which was entropically controlled. The enthalpy and enthropy positive values resulted from melting of “icebergs” or “flickering clusters” around the surfactant, leading to increased packing of hydrocarbon chains within the micellar core in a non-random manner. This can be possibly explained by caffeic acid governing the 3D matrix structure of water around the micellar aggregates. The fact that both enthalpy and enthropy were positive testifies to the importance of hydrophobic interactions as a major driving force for micellization. Preservatives for storage of postharvest products facilitate micellar association, thereby extending their service life without the need to use increased amounts of additives. According to Laguerre et al. [32], the deeper the phenolic is in the micelle, the less antioxidant the phenolic. If a surfactant not allowed as an ingredient or food additive is to be used, then carefully washing it off before consuming the product can avoid its potentially adverse impact on health. 

## Figures and Tables

**Table 1:** CMC (mM kg^−1^) and mean degree of micellization (β) in the SDS–CA micellar system at variable mole ratios as calculated from electrical conductivity plots. The uncertainly values are below 10%.

[CA] × 10^−5^ mol kg^−1^	Temperature (°C)													
	25		30		35		40		45		50		55	
	CMC	β	CMC	β	CMC	β	CMC	β	CMC	β	CMC	β	CMC	β
30	7.39	0.70	7.13	0.74	7.03	0.77	7.04	0.74	7.03	0.71	6.92	0.75	6.81	0.64
15	7.83	0.77	7.56	0.76	7.41	0.77	7.35	0.60	7.30	0.67	7.15	0.68	7.01	0.57
7.5	8.16	0.66	7.77	0.69	7.58	0.67	7.55	0.73	7.53	0.64	7.37	0.76	7.21	0.57
3.0	8.32	0.74	7.85	0.67	7.67	0.60	7.62	0.68	7.58	0.60	7.41	0.63	7.25	0.66
1.5	8.34	0.79	8.11	0.74	7.92	0.67	7.86	0.64	7.69	0.60	7.62	0.65	7.45	0.61

**Table 2:** ∆*G*^0^*_m_* values (kJ mol^−1^) for SDS–CA micelles as obtained by substituting CMC and β values into Equation (3). The uncertainly values are below 10%.

[CA] × 10^−5^ mol kg^−1^	*T* (°C)						
	25	30	35	40	45	50	55
30	−37.1	−37.9	−38.6	−39.2	−39.9	−40.6	−41.3
15	−36.9	−37.7	−38.4	−39.0	−39.7	−40.4	−41.1
7.5	−36.7	−37.6	−38.3	−38.9	−39.6	−40.3	−41.0
3.0	−36.7	−37.5	−38.2	−38.9	−39.5	−40.2	−41.0
1.5	−36.6	−37.4	−38.1	−38.7	−39.5	−40.1	−40.8

**Table 3:** Δ*H*^0^_m_ (kJ mol**^−^**^1^) and Δ*S*^0^_m_ (kJ mol**^−^**^1^ K**^−^**^1^) for the SDS–caffeic acid system as calculated from the variation of Δ*G*^0^_m_ with *T*. The uncertainly values are below 10%.

[CA] × 10^−5^ mol kg^−1^	Δ*H*^0^_m_ kJ mol^−1^	Δ*S*^0^_m_ kJ mol^−1^ K^−1^	*R*^2^
30	3.9	0.14	0.9992
15	4.2	0.14	0.9992
7.5	4.9	0.14	0.9979
3.0	5.0	0.14	0.9988
1.5	4.7	0.14	0.9988
